# Three-dimensional automatic segmentation of root canals with focus on the second mesiobuccal canal using nnU-Netv2 on CBCT images: deep learning approach

**DOI:** 10.1186/s12903-026-08285-8

**Published:** 2026-04-07

**Authors:** Deniz Meltem Güllü, Kaan Orhan, Nevin Kartal

**Affiliations:** 1 Faculty of Dentistry, Department of Endodontics, İstanbul Health and Technology University, İstanbul, Türkiye; 2 Faculty of Dentistry, Department of Oral and Maxillofacial Radiology, Ankara University, Ankara, Türkiye; 3 Faculty of Dentistry, Department of Endodontics, Marmara University, İstanbul, Türkiye

**Keywords:** Artificial intelligence, Deep learning, Root canal morphology, Second mesiobuccal canal, Maxillary first molar

## Abstract

**Background:**

Artificial intelligence (AI) has the potential to reduce interpretation errors and save time during the evaluation of cone beam computed tomography (CBCT) images. This study aimed to assess the performance of AI in identifying and segmenting the second mesiobuccal canal (MB2), with concurrent segmentation of the main root canals, in the maxillary first molar prior to endodontic treatment.

**Methods:**

In this study, 202 CBCT images that met the inclusion criteria were obtained from an anonymized database provided by Craniocatch (Eskişehir, Türkiye), with no associated personal data. The nnU-Netv2 model implemented with the PyTorch library was used for the detection and three-dimensional (3D) automatic segmentation of root canals. Owing to the narrow structure of the MB2 canal, labels were preprocessed via binary dilation with SciPy (v1.10.1), and training was conducted in two stages by applying different dilation levels. The performance of the artificial intelligence model was evaluated via the confusion matrix and further assessed with additional metrics, including the Dice score (DC), Jaccard index (JI), 95% Hausdorff distance (HD), and area under the curve (AUC).

**Results:**

In this study, the nnU-Netv2 model achieved a sensitivity of 0.538, a precision of 0.719, a DC of 0.616, a JI of 0.445, a 95% HD of 0.874, and an AUC of 0.8 for 3D automatic segmentation of MB2.

**Conclusions:**

This study is the first to apply the nnU-Netv2 model for 3D automatic segmentation of the MB2 canal in untreated teeth and highlights its potential utility in endodontic imaging. Further refinements in these systems may enable rapid and reliable 3D automatic segmentation of MB2 and enhance endodontic treatment quality and patient outcomes.

## Background

A comprehensive understanding of root canal system morphology is essential before performing endodontic treatment, as the success of the procedure is directly influenced by the effective disinfection, shaping, and obturation of all existing root canals [1]. Accurate identification of variations in root canal morphology is critical for treatment success [2]. These variations are particularly significant in maxillary molars, where the number of root canals observed prior to treatment varies substantially [3]. Reports in the literature indicate that missed canals significantly affect the outcomes of failed root canal treatments [4–6]. The maxillary first molar commonly exhibits an additional root canal located in the palatal aspect of the mesiobuccal root. Failure to identify and localize this canal may lead to unsuccessful endodontic treatment [7, 8]. This canal is commonly referred to as the second mesiobuccal canal (MB2) [9]. First described nearly a century ago, the MB2 canal continues to be an important subject in endodontic research [10]. The prevalence of MB2 canals varies widely in the literature, with studies suggesting associations with factors such as age, race, and sex [11–13]. The prevalence of MB2 canals in maxillary first molars has been reported to range from 50% to 90% in different cases [9, 14, 15]. Furthermore, a global study reported that the prevalence of MB2 canals ranged from 48.0% to 97.6% across different regions and that the overall global prevalence was 73.8% [12].

Cone beam computed tomography (CBCT) is the preferred method for visualizing root canal morphology in endodontics, providing precise, virtually instant, and accurate three dimensional (3D) radiographic images. In recent years, CBCT imaging has become an important method for identifying additional canals in teeth [1]. Maxillary molars have a greater incidence of MB2 when preoperative CBCT scans are obtained [16]. However, assessing root canal morphology depends on clinician expertise, which varies considerably in the interpretation of CBCT images. This method may be influenced by personal subjectivity and experience, which could lead to failure to detect root canals. Therefore, the use of artificial intelligence-assisted systems in the evaluation of root canal morphology can provide an objective and standardized approach, offering a fast and reliable solution for the identification of complex root canal systems.3D imaging software is widely used for the automatic segmentation of various organs and their substructures, providing significant benefits in the fields of diagnosis, surgical planning, and simulation [17]. In endodontics, automated segmentation of the 3D pulp space in CBCT images will significantly contribute to diagnosis and treatment planning [18].

Artificial intelligence (AI) refers to the ability of computer systems and machines to perform tasks associated with human intelligence, and methods such as machine learning (ML) are used in this process. Deep learning (DL), a subset of machine learning (ML), has become a key approach in AI [19]. Unlike traditional ML, which depends on manually designed features and task-specific optimizations requiring human expertise, DL methods learn features directly from data. This ability to learn hierarchical representations through multiple processing layers allows DL models to extract and represent data at different levels of abstraction, making them particularly effective for complex AI tasks [20]. In this context, many studies evaluating the automatic segmentation of the pulp space have also employed a deep learning-based U-Net model [21–23]. U-Net is considered a convolutional neural network (CNN) model, but it is a specialized type specifically designed for segmentation tasks in the field of medical imaging. nnU-Net v2 is an advanced framework based on the U-Net architecture that incorporates several key innovations. According to a recent benchmark study, nnU-Netv2 outperforms most other 3D segmentation methods in biomedical imaging tasks (https://link.springer.com/chapter/10.1007/978-3-031-72114-4_47). It is highly effective as a self-configuring deep learning segmentation framework, capable of automatically adapting its preprocessing, network architecture, training, and postprocessing strategies to meet the requirements of each new task in the biomedical domain [24]. To the best of our knowledge, no previous study has investigated 3D automatic segmentation of the MB2 canal via nnU-Net v2. This study aims to develop a deep learning-based algorithm using nnU-Net v2 architecture and to evaluate its performance in 3D automatic segmentation of all root canals in maxillary first molars, with a specific focus on the MB2 canal on axial CBCT images.

## Methods

### Study design

This retrospective study was conducted in accordance with the principles of the Declaration of Helsinki. The study protocol was approved by the Clinical Research Ethical Committee of the Faculty of Medicine, M. U. (decision no. 09.2023.1374). The CBCT volumes used in this study were obtained from a database anonymized by Craniocatch (Eskişehir/Turkey), ensuring that no personal data were included. In this study, the PyTorch-based nnU-Net v2 framework was used for automatic 3D root canal segmentation of maxillary first molar teeth in 202 CBCT images obtained from different digital tomography devices.

### Data acquisition

#### Data collection and labeling

In this study, CBCT volumes of adult patients containing at least one right or left maxillary first molar were included, whereas scans in which the region of interest could not be clearly delineated for 3D root canal segmentation were excluded. Additionally, CBCT volumes containing maxillary first molars that either have deep restorations/crowns close to the pulp chamber or have undergone root canal treatment and contain posts within the root canal were also excluded from the study.

AI models can learn from image data obtained from different devices, which improves their performance across various conditions. Therefore, the CBCT volumes in this study were acquired from multiple devices. Due to the retrospective nature of the study and the use of fully anonymized datasets sourced from multiple third-party devices, specific acquisition parameters (e.g., exact voxel size and FOV) were not consistently available and are therefore not reported. Patient data were exported in digital imaging and communications in medicine (DICOM) format and uploaded to the labeling module of a web-based software (CranioCatch, Eskişehir, Turkey). Segmentation was performed on axial two-dimensional (2D) slices and reconstructed into three-dimensional (3D) masks. Each DICOM image file, being the standardized registration format for 3D images, was considered a separate project.

**Fig. 1:**
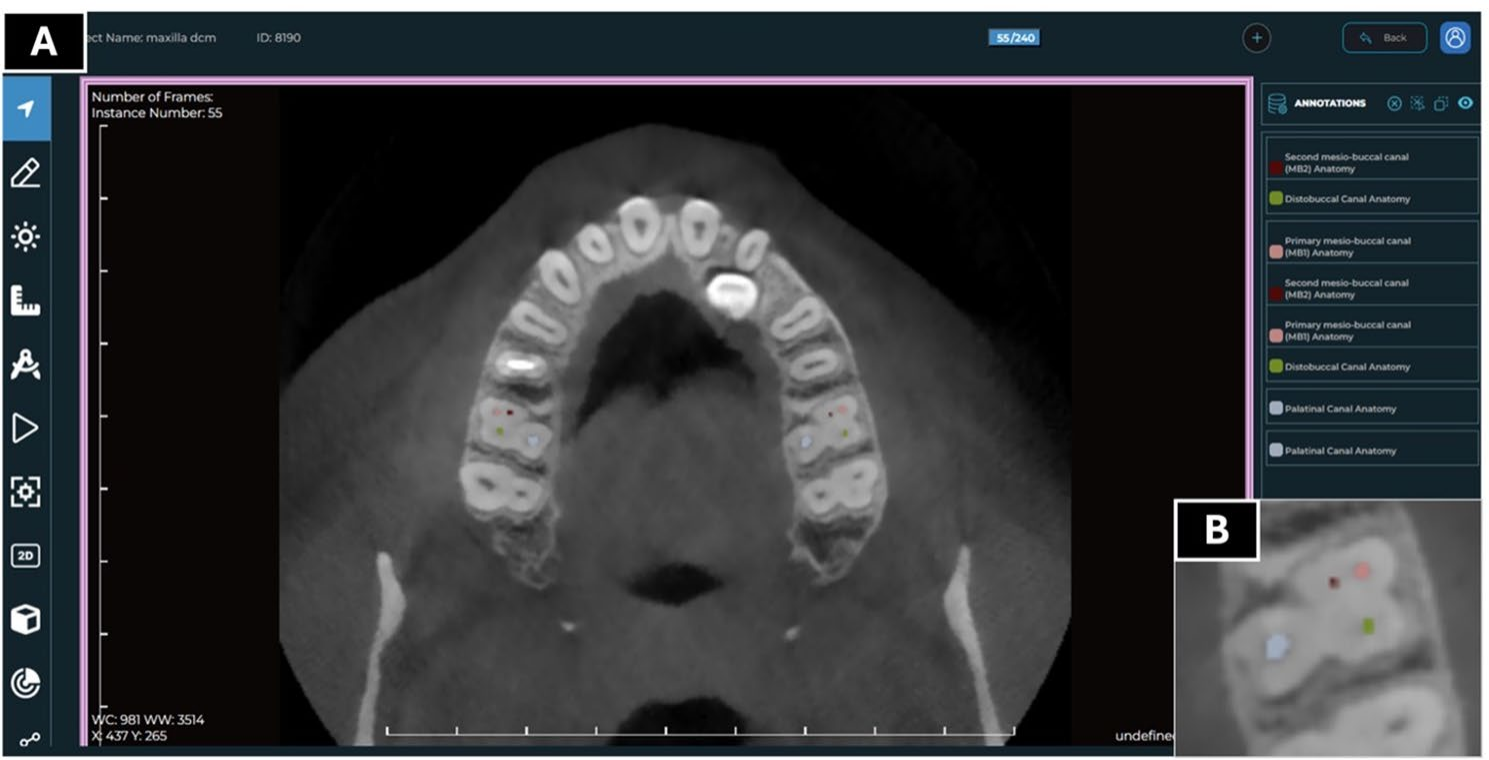
On the labeling module: **(A)** Labeling of MB2 and main root canals in a 3D CBCT image; **(B)** A magnified axial section example showing the labeled MB2 and main root canals

In this study, data labeling was performed via the labeling module of a web-based software (CranioCatch, Eskişehir, Turkey) (Fig. 1A). The root canals were labeled via the polygonal segmentation technique, with axial slices of the CBCT images examined from the pulp chamber floor to the apex (Fig. 1B). For the images with a slice spacing of 0.3 mm, labeling was performed at 1 mm intervals to prevent the similarity between adjacent slices from negatively impacting the model’s performance. To maintain volumetric continuity within the nnU-Net v2 pipeline, intermediate slices between the 1-mm labeling intervals were reconstructed via linear interpolation during automated preprocessing. This 1-mm interval was specifically chosen to minimize data redundancy and prevent high similarity between adjacent slices from biasing the model, while ensuring that nnU-Net’s resampling capabilities could accurately capture the complex root canal anatomy without loss of structural detail. The MB2 canal was labeled separately in all slices where it was detected as an independent canal, starting from the first slice in which it was identified. If the MB2 canal was absent from all axial slices, it was considered nonexistent, and only the main canals were labeled for those teeth. A total of 231 maxillary first molar teeth from 202 CBCT images were analyzed, with a total of 693 main canals (mesiobuccal, distobuccal, and palatal) and 191 mesiobuccal roots labeled with MB2. The root canals were labeled by a senior endodontic resident (D.M.G.) and subsequently verified by a senior endodontist (N.K.) with 40 years of experience.

#### Binary dilation for training data preparation

In this study, the original-sized labels of the root canals could not be recognized by deep learning algorithms because of the complex root canal anatomy. This was particularly true near the apical foramen, where the labels were smaller than one voxel. As a result, full 3D segmentation of the MB2 and main root canals could not be achieved. Therefore, prior to training, the labels were enlarged via the SciPy library (v1.10.1). The scipy.ndimage.binary_dilation function was applied to perform a dilation operation on the labels. This operation expands the pixels surrounding the label in the image, thereby increasing the size of the structures [25]. Technically, this is a morphological operation that uses a specific structuring element to enlarge the labels. This process utilized a 3 × 3 × 3 cubic structuring element with a connectivity of 1, applied across all 3D axes to ensure volumetric consistency. In this study, the method was implemented in two stages. In the first stage, a single iteration of dilation was performed; in the second stage, the dilation was increased to two iterations. This specific strategy was chosen to ensure that the labels for the smaller and more complex MB2 canals were effectively integrated into the training process, thereby enhancing the model’s ability to learn without losing intricate anatomical details. To ensure that this dilation process did not lead to anatomical distortion or the artificial inflation of performance metrics, the operation was applied strictly to the training and validation sets. All final evaluations in the test dataset were conducted using the original, non-dilated clinical ground truth, thereby maintaining absolute anatomical validity in the reported results.

### AI Development

#### Development and training of AI models via deep learning techniques

**Fig. 2:**
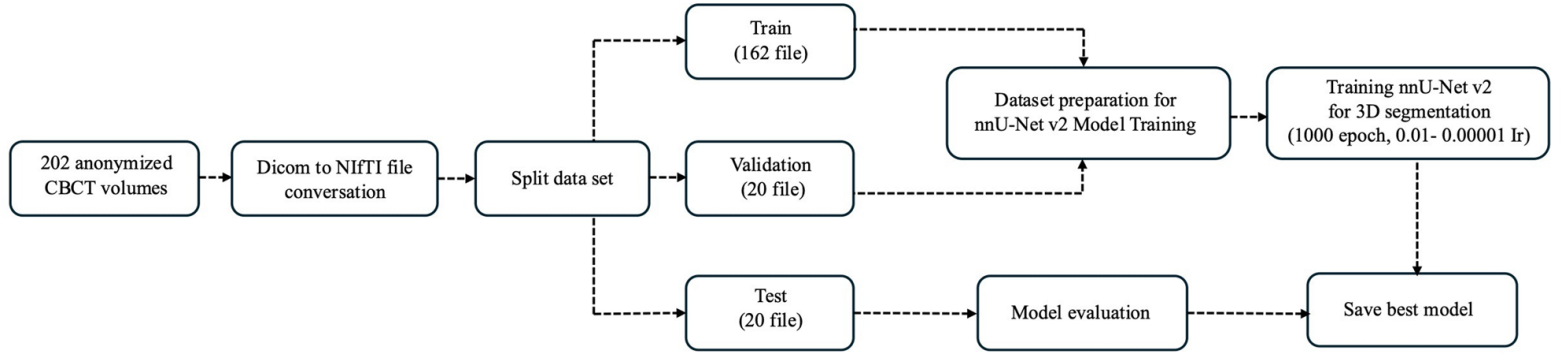
Model pipeline of automatic root canal segmentation

The labeled data were converted from DICOM format to NIfTI (Neuroimaging Informatics Technology Initiative) format in preparation for model training. To ensure that images used in training were not reused for testing, the dataset -comprising 202 CBCT volumes and 231 teeth- was randomly partitioned into three groups at the CBCT volume (project) level: training (80%, *n* = 162 volumes), validation (10%, *n* = 20 volumes), and testing (10%, *n* = 20 volumes). Given the high prevalence of MB2 canals in the study population (191 out of 231 teeth, ~ 82.7%), this random partitioning process naturally ensured a balanced and representative distribution of both MB2-positive and MB2-negative cases across all subsets. Therefore, a separate stratification algorithm was not required to maintain anatomical consistency between the groups. The pipeline used for the automatic segmentation of the MB2 and main root canals is shown in Fig. 2.

The automatic root canal segmentation algorithm, which is based on nnU-Net (v2.1), was developed via the Python programming language (v3.10.12) and the PyTorch library. To maintain the self-configuring advantages of the nnU-Netv2 framework, the standardized “out-of-the-box” pipeline was utilized. This included the application of the default augmentation suite (random rotations, scaling, mirroring, and gamma correction) to enhance model robustness against varying CBCT sensor characteristics. Instead of five-fold cross-validation, a fixed 80/10/10 split was maintained at the project level to ensure a completely independent test set for final performance reporting. To address the extreme class imbalance between the fine MB2 canal anatomy and the background, the previously described two-stage binary dilation strategy was implemented, ensuring the hybrid loss function received sufficient signal from these narrow structures during training.

The model received training for 1000 epochs, starting with a learning rate of 0.01, which was reduced by 0.00001 after each epoch, and the model with the best performance was saved.

The mathematical processing during the model’s training was carried out via a Dell PowerEdge T640 Compute Server (Dell Inc., Round Rock, TX, USA), a Dell PowerEdge T640 GPU Compute Server (Dell Inc., Round Rock, TX, USA), and a Dell PowerEdge R540 Storage Server (Dell Inc., Round Rock, TX, USA).

#### nnU-Net v2 framework

**Fig. 3:**
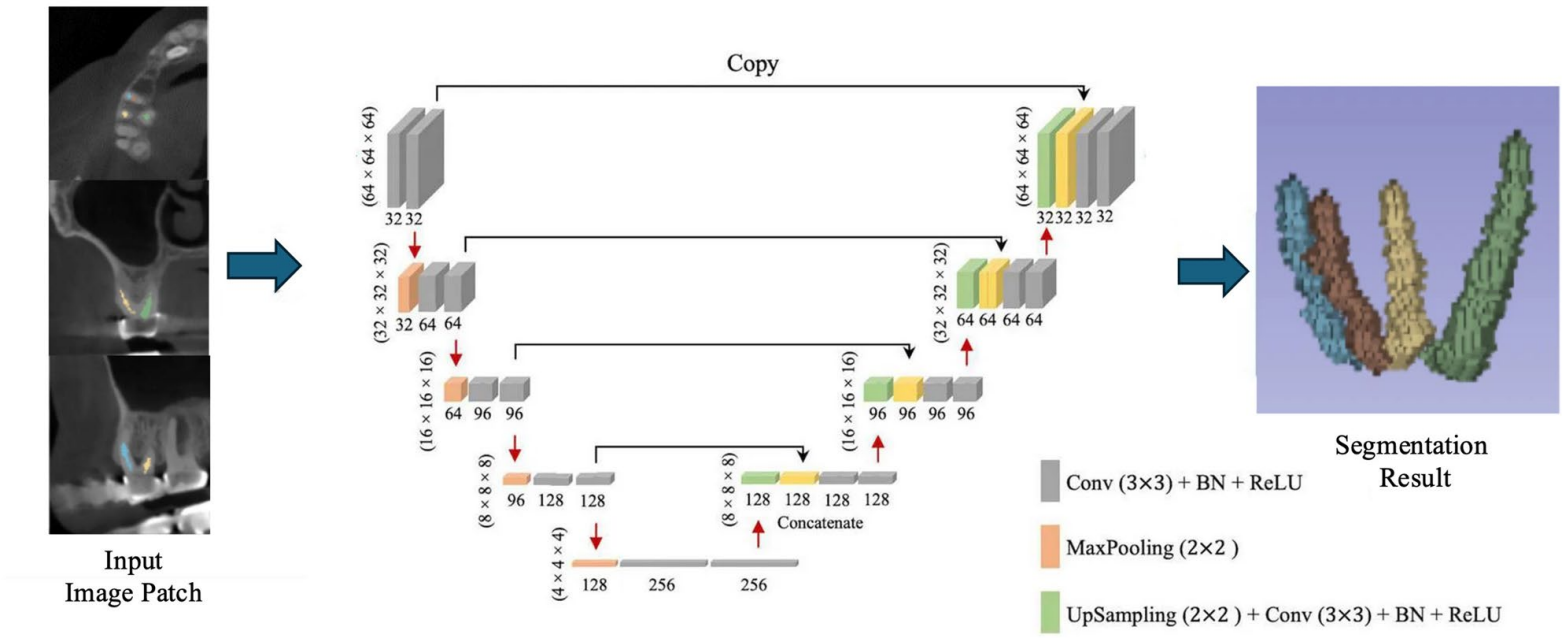
A general overview of the nnU-Net v2 model algorithm used for automatic segmentation of root canals in this study

nnU-Net is a deep learning-based segmentation method that automatically configures itself for any new biomedical task, including data preprocessing, network architecture, training, and postprocessing. This framework is based on three relatively simple U-Net models, each incorporating only minor modifications to the original U-Net [26]. Additionally, nnU-Net is capable of processing both 2D and 3D datasets, thereby enhancing its applicability across various medical imaging modalities (e.g., magnetic resonance imaging, computed tomography). This versatility makes it suitable for segmenting structures such as tumors, organs, and blood vessels. The model automatically adapts to the geometry of the input images. Furthermore, nnU-Net is capable of detailing the steps that can significantly influence model performance, considering various factors surrounding the model. In this framework, U-Net models are automatically configured for each task, and the model with the best performance for the targeted object is selected. This approach enables the automatic selection of the most suitable model for achieving successful segmentation in new tasks. Owing to its ability to adapt dynamically to the characteristics of different datasets, the nnU-Net framework is recommended for state-of-the-art segmentation of medical images [24]. Given these innovative features, a nnU-Netv2-based deep learning method was utilized for the 3D automatic segmentation of root canals in this study (Fig. 3).

#### Assessment metrics

In this study, after the training phase was completed, the model’s performance was evaluated on the test dataset. The test dataset consists of data that the model has not seen before and is crucial for assessing the model’s generalization ability. After the model made predictions on the test dataset, the predictions were compared with the ground truth labels.

**Fig. 4:**
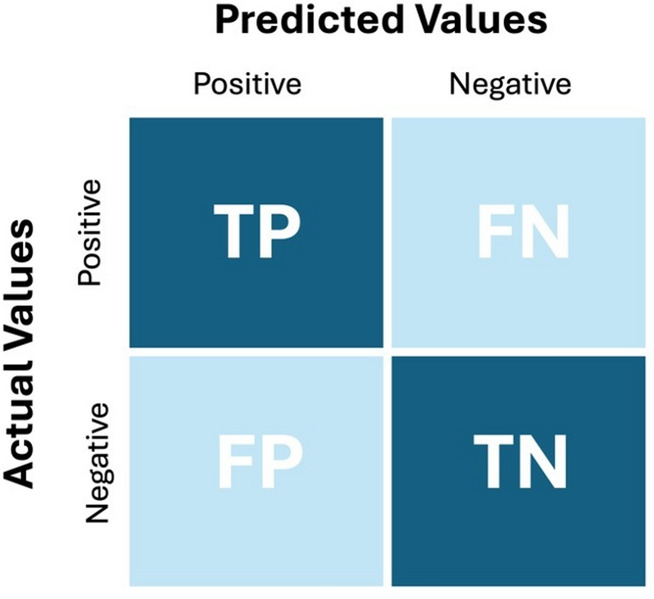
Confusion matrix

Performance metrics were calculated via data from the confusion matrix, which compares the predicted classes with the ground truth classes (Fig. 4).

The following procedures and metrics were used to assess the performance of the deep learning model:

- The rates of true positives (TPs), false positives (FPs), and false negatives (FNs) were calculated.
  - TP : Target structure correctly identified in segmentation.
  - FP : Non-target structures incorrectly identified as the target.
  - FN : Target structures missed or excluded in segmentation.
- The sensitivity (recall) and precision values were subsequently calculated via the TP, FP, and FN values with the formulas provided below.
  - Sensitivity (Recall): TP/(TP + FN)
  - Precision: TP/(TP + FP)
- Additionally, the performance of the developed model in the 3D automated segmentation of MB2 and main root canals was evaluated via the following metrics:
- Additionally, 95% Confidence Intervals (CIs) were calculated for all primary metrics using the Wilson score method for proportions and the standard error for overlap and distance metrics to assess the statistical robustness of the model.

#### Dice score (Dice coefficient – DC)

It is a metric that reflects the degree of overlap between the labeled and predicted regions, reflecting the effectiveness of the segmentation.

$$DC=\frac {(2 \times \mid P \cap T \mid)}{(\mid P \mid + \mid T \mid)} \;\;\;\;\;\;\; P: Predicted\;\;\;T: True$$

#### Jaccard index (Jaccard similarity index, JI)

It is defined as the ratio of the intersection of the labeled and predicted regions to their union.

$$JI=\frac {\mid P \cap T \mid}{\mid P \cup T \mid} \;\;\;\;\;\;\; P: Predicted\;\;\;T: True$$

The Dice score and Jaccard index are metrics used to evaluate segmentation accuracy, with values ranging from 0 to 1. A value of 1 indicates perfect overlap, whereas a value of 0 indicates no overlap. Therefore, as the values approach 1, it indicates an improvement in the accuracy of the segmentation.

#### 95% Hausdorff distance (95% HD)

In the comparison of two sets, it measures only the distance between the farthest points of each set. A lower Hausdorff distance indicates higher accuracy in the segmentation.

$$d_{H95}(A, B) = max (d_{95}(A, B), d_{95}(B, A))$$

- A: The set of points belonging to the ground truth segmentation.
- B: The set of points belonging to the predicted segmentation.

#### Receiver operating characteristic (ROC) analysis

The ROC/AUC values were computed based on voxel-wise probability maps generated by the model. Each voxel’s predicted probability was evaluated against the ground truth labels to construct the ROC curve. This approach allows for a more granular assessment of the model’s confidence in identifying root canal structures across the entire 3D volume.

## Results

**Fig. 5:**
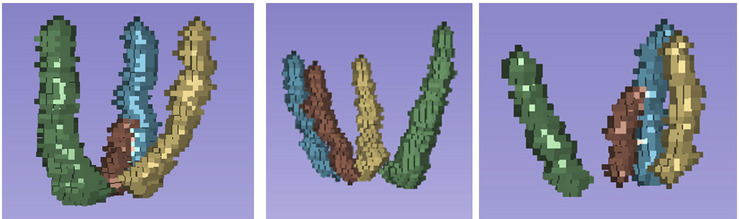
3D automatic segmentation images of MB2 and main root canals in different canal configurations performed with the nnU-Netv2 model (Mesiobuccal-1 : Blue, Mesiobuccal-2: Red, Distobuccal: Yellow, Palatal: Green)

**Fig. 6:**
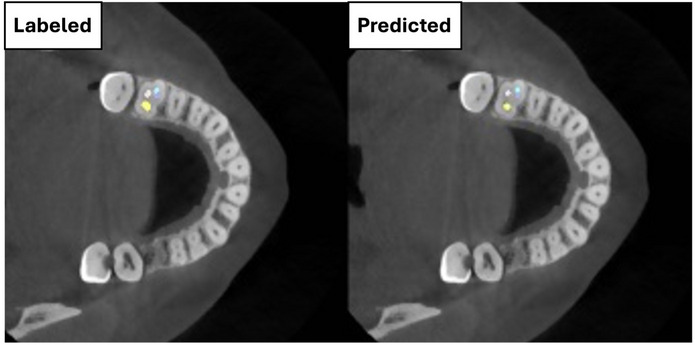
The automatic segmentation of MB2 and main root canals on axial CBCT slices

In our study, 3D automatic segmentation of the MB2 and main root canals of the maxillary first molar was performed via the nnU-Netv2 model (Fig. 5). The segmentation results for the MB2 and main root canals via the nnU-Netv2 framework are similar to the true morphology of the root canals (Fig. 6). Examples of both successful and less accurate segmentations are provided to illustrate variability in model performance.

**Fig. 7:**
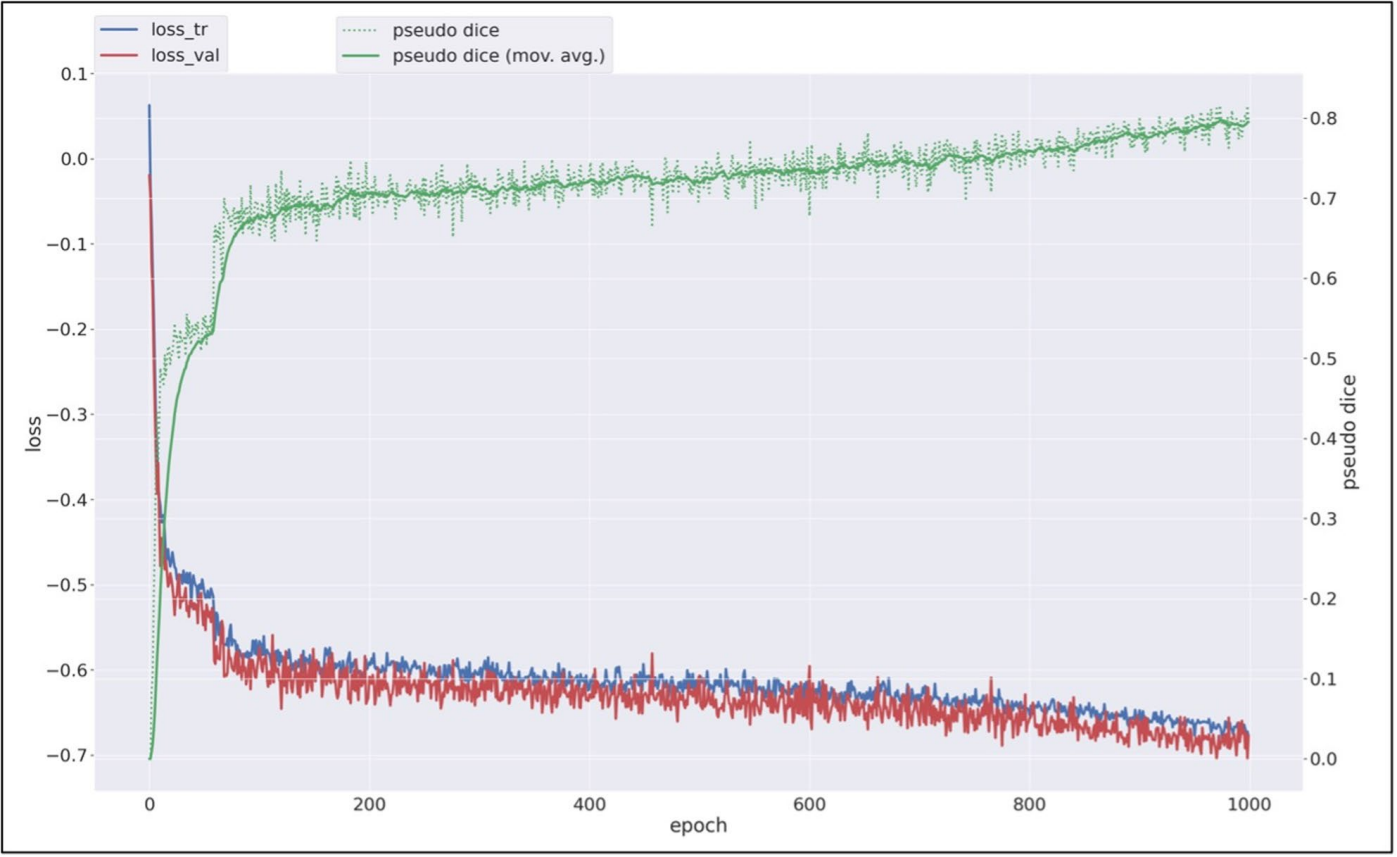
The evaluation of the Dice score and general loss functions during the training process in our study (loss_tr: training set loss curve, loss_val: validation set loss curve, pseudo dice: validation set pseudo dice curve, pseudo dice (mov. avg.): moving average of the validation set pseudo dice curve)

**Fig. 8:**
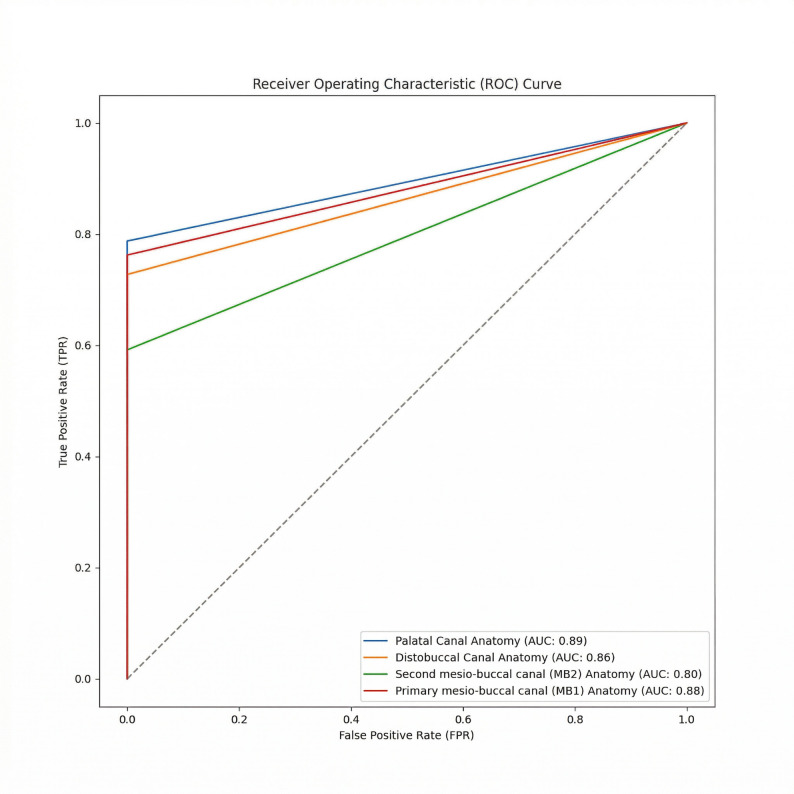
ROC Curve for the Segmentation of MB2 and Main Root Canals Using the nnU-Netv2-based AI Model

The sensitivity and precision values for the MB2 and main root canals of the deep learning model are presented in Table 1. The sensitivity and precision values for the MB2 canal were 0.538 and 0.719, respectively (Table 1). The general loss functions and DC were evaluated over 1000 epochs (training iterations) using the dataset employed in our study (Fig. 7). The DC, JI, and 95%HD values for the MB2 and main root canals are provided in Table 2. The DC, JI, and 95% HD values for the MB2 canal were 0.616, 0.44, and 0.87 respectively. The AUC values for the model’s MB2 and main root canals were computed from the receiver operating characteristic (ROC) curve (Fig. 8). The AUC value for the MB2 canal was determined to be 0.8.

**Table 1:** Sensitivity and Precision Performance Metrics (with 95% CI) for the Automatic Segmentation of MB2 and Main Root Canals Using the nnU-Netv2 Model

Metrics	MB2	MB	DB	*P*
Sensitivity	0.538 (0.47–0.61)	0.721 (0.65–0.79)	0.617 (0.54–0.69)	0.654 (0.58–0.73)
Precision	0.719 (0.65–0.79)	0.726 (0.66–0.79)	0.781 (0.72–0.84)	0.817 (0.76–0.87)

*MB2* Second Mesiobuccal Canal, *MB* Mesiobuccal Canal, *DB* Distobuccal Canal, *P* Palatal Canal

**Table 2:** DC, JI, and 95% HD Values (with 95% CI) for the Automatic Segmentation of MB2 and Main Root Canals Using the nnU-Netv2 Model

Metrics	MB2	MB	DB	*P*
DC (Dice)	0.616 (0.55–0.68)	0.723 (0.66–0.78)	0.690 (0.63–0.75)	0.727 (0.67–0.78)
JI	0.445	0.567	0.526	0.571
95%HD	0.874	0.9	0.99	1.23

*MB2* Second Mesiobuccal Canal, *MB* Mesiobuccal Canal, *DB* Distobuccal Canal, *P* Palatal Canal

## Discussion

Artificial intelligence (AI) has the potential to emulate human intelligence in making complex and predictive decisions in healthcare, and its application in medical image analysis is rapidly expanding [27]. In particular, deep learning methods have gained increasing importance in medical image processing and are highly effective in handling large and complex images. This growing trend in healthcare is mirrored in the field of endodontics, where AI technologies are also finding numerous applications. Among the key applications of AI in endodontics are the detection and diagnosis of periapical lesions, determination of working length, assessment of the morphology of the root and root canal system, and identification of previously performed canal treatments [28–31]. In particular, it is believed that the use of artificial intelligence in evaluating root canal morphology will provide clinicians with a safer and faster method, offering valuable support in their decision-making process.

In a previous study, a deep learning system applied to panoramic radiographs demonstrated a diagnostic accuracy of 86.9% in detecting single or multiple distal roots in mandibular first molars [30]. In another study, an algorithm developed by artificial intelligence demonstrated the ability to accurately measure the 3D modification of root canal curvature after instrumentation [32]. Additionally, a study compared three different deep learning architectures (Residual U-Net, XceptionU-Net, and U-Net) for the detection and classification of C-shaped canal anatomy [22]. The best results were reported with XceptionU-Net. Moreover, a limited number of deep learning studies have focused on the segmentation of the pulp chamber and root canals. In a study conducted on crown and root canal segmentation, the U-Net deep learning architecture was employed, yielding an average accuracy of 0.99, a sensitivity of 0.82, an F1 score of 0.73, and an AUC of 0.91 [21]. In another study, the U-Net model trained on CBCT image data successfully performed segmentation of the teeth and pulp chamber, achieving a Dice score of 98.59%, and it was reported that it could be a useful method for acquiring morphological information [33].

However, in addition to these studies on root canal morphology, to the best of our knowledge, only a limited number of studies have evaluated the detection of MB2 using deep learning methods on patient images. In a recent study, segmentation of untreated MB2 canals missed during the diagnostic phase in fully treated maxillary molars was performed via artificial intelligence, employing the U-Net architecture [34]. The sensitivity value for MB2 detection performance was 0.8, the specificity was 1, the positive predictive value was 1, and the negative predictive value was 0.83. Furthermore, the deep learning method’s MB2 segmentation performance was evaluated via a metric, which yielded an average value of 0.3018 for the test set. While these results provide valuable insights, our study introduces a novel methodological approach using nnU-Netv2 for 3D CBCT segmentation in untreated teeth.

In another study, two-dimensional annotations were made on axial slices of CBCT images of the maxillary first molars and/or maxillary second molars, and the YOLOv5 architecture was used for model training [35]. In this study, the segmentation model for the MB2 canal demonstrated a sensitivity value of 0.92, a precision value of 0.83, and an F1 score of 0.87. The area under the receiver operating characteristic (ROC) curve (AUC) was reported as 0.84. Additionally, the model achieved a mean average precision (mAP) of 0.88 at an intersection over union (IoU) threshold of 0.5. On the basis of these results, the authors suggest that the MB2 canals of maxillary molars can be detected and segmented in CBCT images via deep learning-based artificial intelligence systems. In the present study, three-dimensional (3D) annotations were made on CBCT images of maxillary first molars, and the performance of the nnU-Net framework was evaluated for segmenting the MB2 and main root canals. The model is a customized and more advanced variant of the U-Net architecture and is designed for a wide range of segmentation tasks in medical imaging. It can be further adapted for specific applications, such as root canal segmentation, and has demonstrated satisfactory results in our study. To the best of our knowledge, our study is the first to assess the 3D automatic segmentation of the MB2 canal in untreated teeth via nnU-Net v2.

This study has several limitations. While a rigorous, hierarchical verification process was maintained by an experienced endodontist to ensure high-quality ground truth, the study is limited by the absence of formal, independent inter-observer and intra-observer reliability analyses. However, the consensus-based approach adopted here aimed to minimize individual subjectivity and prioritize anatomical precision during the labeling phase. Future research incorporating multiple independent blinded observers could provide further validation of the consistency and reproducibility of these protocols.

The moderate sensitivity (0.538) for MB2 detection reflects the inherent difficulty in segmenting structures that often approach or fall below the 0.3 mm voxel resolution of typical CBCT scans. An error analysis of failed segmentations revealed that failure typically occurred in cases with extreme canal calcification or high levels of anatomical noise, where the model’s confidence was lower.

The fine and narrow anatomy of root canals presents significant challenges for artificial intelligence systems in terms of accurately defining and segmenting these structures. In this context, the training process could not be completed because of the inherently small size of the root canal labels, and as a result, the model’s performance could not be meaningfully evaluated. In the first phase of our study, a single-level dilation process was applied to the original labels. Consequently, performance metrics for the nnU-Netv2 model were calculated for the segmentation of MB2 and the main root canals. In this phase, significant findings were obtained for the main canals of maxillary first molar teeth. However, owing to the smaller volume of the MB2 canal than of the main root canal, the application of a single-level dilation process to the labels of this canal was insufficient for the AI system to recognize it and integrate all the data into the training process. In the second phase of our study, a two-level dilation process was applied to the original labels. With this adjustment, all labeled data for the MB2 canal were successfully recognized by the AI system, and a meaningful training process was effectively carried out. In future studies, with the development of advanced deep learning techniques, the challenges in the 3D automatic segmentation of small structures, such as root canals, could be overcome.

## Conclusion

This study presents, for the first time, the use of a deep learning-based nnU-Netv2 framework for 3D segmentation of root canals, with special emphasis on the MB2 canal in untreated maxillary first molars. While the results show potential, the moderate sensitivity (0.538) and Dice score (0.616) suggest that the system currently serves as a clinical decision-support tool to assist endodontists in identifying complex anatomy, rather than a standalone diagnostic replacement.

## Data Availability

The original contributions presented in the study are included in the article. Further inquiries can be directed to the corresponding authors.

## Abbreviations

AI: Artificial Intelligence
CBCT: Cone Beam Computed Tomography
3D: Three Dimensional
MB2: Second Mesiobuccal Canal
HD: Hausdorff Distance
DC: Dice Coefficient
JI: Jaccard Index
AUC: Area Under the Curve
ML: Machine Learning
DL: Deep Learning
CNN: Convolutional Neural Network
DICOM: Digital Imaging and Communications in Medicine
2D: Two-Dimensional
NIfTI: Neuroimaging Informatics Technology Initiative
TP: True Positive
FP: False Positive
FN: False Negative
ROC: Receiver Operating Characteristic
MB: Mesiobuccal Canal
DB: Distobuccal Canal
P: Palatal Canal
